# Replacement, Reduction, and Refinement of Animal Experiments in Anticancer Drug Development: The Contribution of 3D In Vitro Cancer Models in the Drug Efficacy Assessment

**DOI:** 10.3390/biomedicines11041058

**Published:** 2023-03-30

**Authors:** Elena M. Tosca, Davide Ronchi, Daniele Facciolo, Paolo Magni

**Affiliations:** Dipartimento di Ingegneria Industriale e dell’Informazione, Università Degli Studi di Pavia, 27100 Pavia, Italy; elenamaria.tosca@unipv.it (E.M.T.);

**Keywords:** 3D in vitro cancer models, spheroids, patient-derived organoids, drug efficacy assessment, preclinical-to-clinical translation, 3R principles of animal experiments, mathematical modeling and simulation

## Abstract

In the last decades three-dimensional (3D) in vitro cancer models have been proposed as a bridge between bidimensional (2D) cell cultures and in vivo animal models, the gold standards in the preclinical assessment of anticancer drug efficacy. 3D in vitro cancer models can be generated through a multitude of techniques, from both immortalized cancer cell lines and primary patient-derived tumor tissue. Among them, spheroids and organoids represent the most versatile and promising models, as they faithfully recapitulate the complexity and heterogeneity of human cancers. Although their recent applications include drug screening programs and personalized medicine, 3D in vitro cancer models have not yet been established as preclinical tools for studying anticancer drug efficacy and supporting preclinical-to-clinical translation, which remains mainly based on animal experimentation. In this review, we describe the state-of-the-art of 3D in vitro cancer models for the efficacy evaluation of anticancer agents, focusing on their potential contribution to replace, reduce and refine animal experimentations, highlighting their strength and weakness, and discussing possible perspectives to overcome current challenges.

## 1. Introduction

Cancer is one of the major leading causes of human death, and oncology represents the largest therapeutic area in the pharmaceutical industry in terms of number of projects, clinical trials and research investments. The development of new anticancer agents is a complex, time-consuming and expensive process that is associated with an high attrition rate [1,2]. The typical development process of (anticancer) drugs includes a preclinical phase followed by three clinical phases. Currently, the preclinical studies required by regulatory authorities are mainly based on bidimensional (2D) cell cultures and animal models, which remain one of the pivotal experimental approaches of translational cancer research [3].

In 2D in vitro experiments, cell cultures are exposed to drug candidates, usually at constant concentrations for a fixed period. The aim is to screen and rank a large number of test compounds, assessing if they have an anticancer effect, typically quantified by measuring cell viability. Other endpoints, such as target engagement and downstream effects, can also be assessed as part of these studies to provide information on the potency and on the mode of action of the candidates. Compounds that show promising efficacy profiles in 2D in vitro systems progress to in vivo tests in animal models. Despite 2D cell cultures are the most commonly used in vitro models for drug screening due to their easy handling, reproducibility and low cost, they are unable to fully reproduce the properties of in vivo solid tumors [4,5]. Indeed, in vivo, tumors grow in three-dimensional (3D) conformation with a specific organization and architecture that is not modeled in 2D cell cultures. Growing in a 2D plastic substrate, tumor cells have equal and unlimited access to nutrients and oxygen and are uniformly exposed to drug treatment. Consequently, numerous processes, such as diffusion-limited distribution of oxygen, nutrients and metabolites as well as drug penetration, are lost in 2D cell cultures. In addition, the physical organization in monolayers greatly limits cell–cell interactions, which are responsible for cell differentiation, proliferation, vitality, expressions of genes and proteins, drug metabolism and other cellular functions. This leads to higher proliferation rates compared to in vivo cancer cells, which often results in higher drug sensitivity. As a result, the capabilities of 2D cell cultures of predict anticancer drug efficacy is impaired [6,7] and a large number of agents (also showing low efficacy) proceed to the subsequent in vivo phase, which contributes to an overuse of animals, thereby increasing the overall length and cost of the drug development process [8,9].

In in vivo experiments, the selected compounds are administered to animal models to investigate the pharmacokinetics (PK) and to assess efficacy and safety. In this context, ectopic xenografts are the most popular animal models [10]. They consist of immunosuppressed animals, usually mice or rats, in which human cancer cells are inoculated subcutaneously (s.c.) in the flank. Alternative and more complex xenograft models, differing in the transplant site (orthotopic xenograft) or in the source of tumor cell lines (syngeneic xenograft or patient-derived xenograft, PDX), have been well-established, too [11]. Xenografted animals are divided into several arms receiving placebo or anticancer agents following different administration protocols. Anticancer activity is typically assessed by monitoring tumor volume over time and computing tumor growth delay and/or tumor growth inhibition (TGI) as efficacy metrics. The investigated compounds are ranked according to the efficacy assessment, and precedence is given to agents showing the greatest antitumor activity. However, the aim of animal experiments is not only to discriminate “effective/not-effective” agents but also to anticipate the concentration levels and the doses which are expected to exert a therapeutic effect in patients. Indeed, for compounds that progress to the clinical phases, drug concentration levels showing a certain activity in a panel of xenograft models are typically used to identify a range of target concentrations in humans, thereby contributing to the dose selection for the First-In-Human (FIH) studies.

Despite the undeniable importance of animal models and their cornerstone role in translational cancer research, they have some serious drawbacks. Promising results from preclinical animal studies are often not confirmed in cancer patients, and candidate agents effective in animal models do not proceed to clinical development, due to the different genetic, cellular and immunological characteristics of animals compared with humans [12,13]. Further, xenograft experiments are very resource- and time-consuming and are encumbered with ethical/regulatory limitations. Indeed, replacing, reducing and refining (3Rs) animal experiments in scientific research has become an international priority, and regulatory agencies and industry are working toward a decrease in animal use [14].

3D in vitro cancer models are emerging as a promising method to bridge the gap between 2D in vitro cell cultures and animal models, due to their ability to more faithfully mimick in vivo tumors [9,15,16]. Various 3D in vitro cancer models have been developed, including spheroids and organoids, which differ in terms of tumor cell sources, culture protocols and time required for establishment [16]. They have distinct and overlapping purposes, among which are studies in cancer biology, drug screening, anticancer efficacy assessment as well as personalized medicine.

In this review, we describe the state of the art in the use of 3D in vitro cancer models for the evaluation of drug anticancer efficacy. We will focus on the two most common 3D in vitro models used in cancer research, i.e., spheroids and organoids. For both of them, methodologies for generation, techniques for the assessment of anticancer activity, translatability to the in vivo setting and examples of applications in the drug development will be presented. We will highlight strengths and weaknesses of spheroids and organoids as tools for the anticancer efficacy assessment. Finally, we will discuss the possible contribution of such cancer models to replace, reduce and refine the use of animal models in anticancer drug development, as well as the need to exploit mathematical modeling and simulation (M&S) to reach this goal.

## 2. Types of 3D In Vitro Cancer Models

3D in vitro cancer models are regarded as a promising alternative to animal models, due to their ability to mimic several features of in vivo tumors such as natural tumor architecture, cell–cell interactions, nutrient and oxygen gradients, drug penetration and resistance and, with a varying degree of faithfulness, tumor microenvironment (TME). TME consists of malignant cells, non-malignant cells (cancer-associated fibroblasts, stem cells, endothelial cells and immune cells) and non-cellular components (extracellular matrix, cytokines, chemokines and growth factors), and plays a crucial role in tumor development and progression [17].

**Figure 1:**
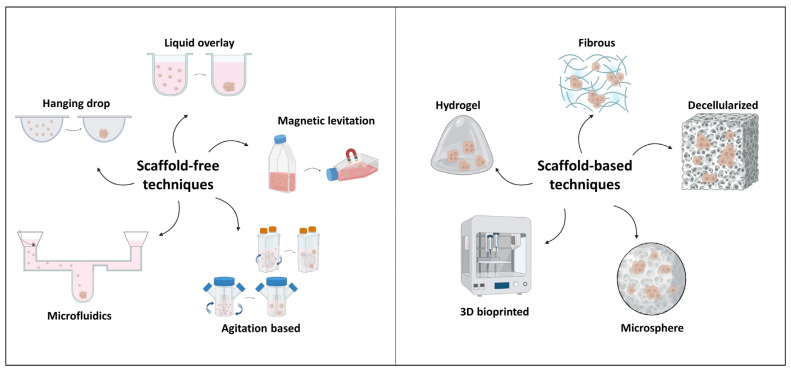
Scaffold-free and scaffold-based techniques for 3D in vitro cancer model generation.

Several methods to assemble 3D in vitro cancer models have been developed (see Table 1 and Table 2 and Figure 1). They are usually categorized into scaffold-free and scaffold-based systems in accordance with the presence or not of a support for cell culture. Scaffold-free (or liquid-based) models include all the systems for which no external artificial platforms are used to promote or induce cell growth and aggregation. In these systems, the 3D architecture is obtained through a cellular self-assembling, in which cancer cells synthesize their own extracellular matrix (ECM), allowing for natural modeling of cell–matrix interactions [18]. Up to now, there are five main scaffold-free techniques, i.e., agitation-based [19,20,21], hanging drop [22,23], liquid overlay [24,25], magnetic levitation [26,27,28] and microfluidic techniques [29,30] (see Table 1). In scaffold-based 3D systems, cell cultures are developed on exogenous structures, made of synthetic or naturally derived polymers, which provide a support for cell growth and mimic ECM conditions. Improving scaffold properties allows optimizing the exchange of nutrients, gasses and waste materials of cancer cells, thus creating conditions similar to those in vivo tumors. In relation to the geometry and production technique, there are different types of scaffolds (see Table 2): hydrogels (such as Matrigel) [31,32], decellularized scaffolds [33,34], fibrous scaffolds [35,36], microsphere scaffolds [37,38] and 3D bioprinted scaffolds [39,40]. 

In the following sections, we will focus on spheroids and organoids, the two most common and versatile 3D in vitro cancer models, highlighting differences and summarizing culture methods.

### 2.1. Spheroids

Spheroids are the simplest 3D in vitro cancer model consisting of spherical aggregates of tumor cells that are either self-assembling or forced to aggregate. Structure and morphology of spheroids are influenced by a multitude of factors, which include cell types, culture methods and media, cell seeding density and mechanical stress. Generally, spheroids with a diameter > 500 µm exhibit similar properties to in vivo avascular tumors, such as heterogeneous cell populations and pathophysiological gradients. Indeed, large spheroids are typically characterized by an external proliferating layer, a middle quiescent layer and an inner core of hypoxic and necrotic cells, caused by the limited distribution of oxygen, nutrients and metabolites in these areas. Spheroid volume generally follows an S-shape growth pattern over time with an initial exponential phase followed by a linear one and then a plateau [59].

Spheroids exhibit strong cell–cell interactions that are enforced by the secretion of ECM proteins. These cell–cell and cell–ECM interactions significantly affect cancer cell proliferation, survival and response to therapy. Indeed, they increase spheroid density, forming a physical barrier that prevents and limits the transport of drugs into the spheroid mass. All these properties strongly influence the therapeutic effects of drugs, increasing drug resistance and improving the reliability of drug screening in cancer spheroids [8].

Spheroids can be divided into several sub-categories. However, the terminology used to indicate the different types of spheroid models is confounding and inconsistent through the literature [9,60,61]. As a strict classification is out of the scope of this paper, we only discern between cell-line derived spheroids, i.e., spheroids generated from immortalized cancer cell lines, and patient-derived spheroids, which are obtained from primary tumor tissue [62].

Immortalized cancer cell lines have often acquired genetic modifications during the immortalization process. Consequently, cell line-derived spheroids are generally easier to handle but less representative of the human native tumors. Hundreds of cancer cell lines from different tumor types have been tested for spheroid formation, showing different degrees of efficiency [42,59,63,64]. For example, Selby et al. explored the possibility of generating spheroids from the 60 cancer cell lines present in the National Cancer Institute (the NCI-60 panel) using ultra-low attachment (ULA) plates, optimizing cell seeding density to obtain a structure of a prespecified diameter (300–500 μm) and providing a classification of generated spheroids based on their morphological characteristics, i.e., the degree of intercellular adhesion [63].

In contrast, patient-derived spheroids maintain the histological and genetic characteristics of the original tumor, and, thus, better recapitulate the inter-individual variability of cancer biology and treatment response observed in cancer patients. However, they exhibit several limitations common to other patient-derived in vitro models, such as variable establishment rates and limited lifespans [62]. Spheroids were successfully grown with varying success from primary tissue of brain [65], breast [66,67,68], colorectal [69], lung [70], ovarian [47,71], uterine endometrial [72] and prostate [73] cancers, and used to test efficacy of anticancer agents, also in the context of personalized medicine.

Further, we can distinguish homotypic spheroids, i.e., cultures of only one type of tumor cells, from heterotypic spheroids, i.e., co-cultures of tumor and stromal cells, such as cancer-associated fibroblasts (CAF) [68] or their precursors [74,75], cells of the immune system [62,76,77] and endothelial cells [78]. Heterotypic spheroids are of great relevance to mimic the cellular heterogeneity of solid tumors and the drug resistance mediated by tumor-stromal cell interactions [8,79,80,81]. In addition, they provide a useful tool for testing new immuno-oncological agents [62,76,77] or novel compounds targeting the stromal components [74,75]. However, co-cultures enhance the complexity of the in vitro model and require optimization of the tumor-stromal cell ratio to correctly recapitulate specific tissue composition as well as of the media components to adequately support growth of both cell types [61].

Spheroids can be obtained after 1 to 7 days of culture, depending on the cells and culture methods. Different approaches are used to generate spheroids, including both scaffold-free and scaffold-based methods (see Table 1 and Table 2). The choice of the generation technique to use is extremely important and is influenced by several factors, such as the research question to address, the used cancer cell type, the expertise of the research team as well as the available equipment and budget. For example, the liquid overlay and the hanging drop techniques are easy and cheap to operate and do not require specialized equipment, thus being compatible with high-throughput drug screening [82,83,84]. Other methods, such those based on microfluidic or bioprinting techniques, may require specialized equipment and expertise that may not be available in all research laboratories [85]. Cancer cell type is a determining factor in the choice of the spheroid formation method. For example, the hanging drop method or the liquid overlay technique in ULA plates might be inadequate to form spheroids from cells such as the Panc-1 and Mia-PaCa pancreatic cancer cells or the CAKI-I renal cancer cells, with a low ability to self-aggregate [63]. For these cell types, alternative methods, for example based on co-culture with supportive cells or embedment in a 3D matrix, such as a hydrogel, may be more appropriate.

Finally, it has been demonstrated that spheroids from the same cancer cell lines can exhibit different properties and responses to therapy depending on the technique used for their generation [64]. This observation is of great relevance, especially because well-established guidelines for spheroid generation are missing and culturing protocols can vary significantly across spheroid studies also involving the same cancer cells. As a result, a high heterogeneity in the spheroid size, shape and cell density, as well as in the cell differentiation degree and physiological behavior is generally observed, which can affect the reliability of study results. Establishing standardized and reproducible protocols for spheroid formation is, therefore, essential to increase uniformity and reproducibility of results across multiple spheroid studies [59].

### 2.2. Organoids

Organoids are complex self-organizing and self-renewing 3D in vitro cultures derived from embryonic stem cells, induced pluripotent stem cells or adult stem cells (ASCs) [86]. The possibility of growing organoids from ASCs originated in 2009 with the seminal work of the Clevers group [87] and was subsequently introduced to cancer research [88]. This paved the way to generate organoids from patient-derived tumor tissue, which generally contains a large number of ASCs. Since then, cultures of patient-derived organoids (PDOs) were established for a multitude of primary and metastatic cancers, including prostate [89], colorectal [90], pancreatic [91], liver [92], breast [93], bladder [94], gastric [95], esophageal [96], lung [97] and ovarian [98] cancers. 

Cancer PDOs are generally derived from tumor tissue specimens directly obtained from patients via biopsy or surgical resection. The success rate of organoid establishment varies significantly among cancer types (see Table 3), ranging from 70–100% for colorectal cancer (CRC) to 15–20% for prostate cancer [99,100]. The derivation time can be significantly different, too, taking up weeks to months. Before being cultured, tumor tissue from human donors has to be first cut into small fragments and then undergo (mechanical or enzymatic) digestion processes. Different techniques for processing tumor fragments as well as different protocols for cancer PDO culture have been proposed. Classically, cell suspensions are embedded within a suitable support that provides an ECM facsimile and a medium containing a cocktail of growth factors. In the vast majority of the studies, cancer organoids are cultured in hydrogel-based scaffolds, typically involving the use of the animal-derived Matrigel, a protein mixture secreted by mouse sarcoma cells. The use of Matrigel for organoid culture is well described and protocols are available [101]. However, due to its natural origin, the Matrigel composition is affected by batch-to-batch variability that might impair the quality control, reliability and reproducibility of organoid studies. Additionally, Matrigel contains animal growth factors that might influence PDO culture and reduce the similarity with the human physiological settings. Alternatively, synthetic hydrogels, which are fully defined and growth-factor-free, can be used [102]. For example, Mosquera et al. demonstrated that synthetic hydrogel-based ECMs can regulate the growth and activity of prostate cancer organoids in a way that is different from that of Matrigel, significantly impacting organoid response to therapeutic drugs [103]. Recently, the possibility of using scaffold-free approaches for organoid cultures has been investigated. For example, organoids have been formed in suspension cultures using ULA plates [44] or through agitation-based techniques [41].

Cancer PDOs display a tumor-like cellular morphology, with typically multiple polarized epithelial structures, that more faithfully mimics the original tumor architecture and functionality than spheroid models [108,109]. Consistent with the primary tumors, cancer PDOs exhibit a range of morphological phenotypes and different cellular architectures [110]. Cohesive organoids are characterized by the presence of multiple structures of different sizes, which can be dense and solid or hollow and cystic, as well as spherical or more irregular in shape. Differently, discohesive organoids form solid cell clusters, some of which have a loose aggregation, giving a “grape-like” appearance. This morphological heterogeneity recapitulates the histological features of the original patient tissue and tumor subtype, but it is also affected by extrinsic factors, such as various oxygenation levels or the composition of the ECM.

An intrinsic limitation of the ASC-derived cancer organoids is that they are composed almost solely of tumor epithelial cells and do not include non-neoplastic stroma components, especially from the immune system. Incorporating TME components into cancer organoid models could be of great relevance especially for some cancer types, such as pancreatic ductal adenocarcinoma (PDAC), which normally includes up to 90% of the stroma component in the tumor mass. To fill this gap, two culturing strategies have been developed [16,111]. In the first approach, the TME is reconstructed by co-culturing well-established organoids with exogenous stroma cells, such as native or reconstituted autologous CAFs or different types of immune cells [109,112]. For example, Tsai et al. generated co-cultures of PDAC organoids with autologous CAFs and T-cells [109]. Additionally, Luo et al. successfully established a co-culture system of CRC PDOs and CAFs by embedding both cell types in a hydrogel-based matrix [113]. Characterization of this in vitro system demonstrated that the co-culturing was able to promote the growth of cancer PDOs, recovering biological pathways that are absent in the conventional PDO cultures but present in patient tissues. The second approach aims to generate cancer organoids that preserve the endogenous TME by culturing tumor epithelium together with endogenous stromal and immune cells as a cohesive unit without the need of reconstitution [111]. This can be achieved through the air-liquid interface (ALI) method, in which minced primary tissue fragments, containing both tumor and stromal cells, are embedded in a collagen matrix within an inner transwell dish, where the top of the collagen gel is exposed to the air, allowing the cells access to oxygen [114]. Alternatively, 3D microfluidic devices can be used to construct organotypic tumor spheroids in collagen hydrogels that retain the native immune cells [115].

**Figure 2:**
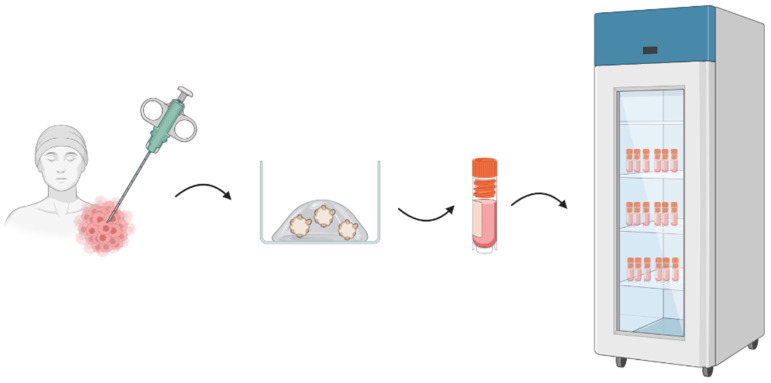
Schematic representation of development of a living biobank containing cancer PDOs.

An attractive feature of cancer PDOs is that, as the traditional cancer cell lines, they can be passaged, cryopreserved, shipped frozen in vials across the world, thawed, and quickly restored to a proliferative culture (Figure 2). Consequently, since the first collection of well-established PDOs were reported in 2015 [90], several living biobanks of cancer and matched healthy PDOs have been established [41,51,93,95,104,116,117]. Across these studies, it was demonstrated that PDOs preserve the histological and genetic diversity of the original tumors, even after long-term culture [118]. This faithful representation has been reported for a multitude of cancer types [95,97,98,105,106], demonstrating that PDOs can provide a useful tool to explore both inter- and intra-tumoral heterogeneity. In this regard, it is important to highlight that cancer PDOs are generally derived from single biopsies or small fragments of surgically resected tissue and, therefore, might not fully encompass the original tumor diversity [100]. 

Further, PDOs can be orthotopically or s.c. transplanted into immunodeficient mice [91,94,98,107,119]. In a reciprocal fashion, organoids can be derived from PDX mice [120,121,122], taking advantage of the already available large PDX libraries. However, the generation of PDX-derived organoids (PDxOs) could be difficult due to the presence of mouse stromal components in the tumor samples that have to be adequately removed. Guillen et al. were able to derive 40 PDxO lines from breast cancer PDXs with a success rate of 85% and cultured them for more than 200 days [121]. Genomics analysis of several PDxO lines revealed high concordance with the original human tumors, also after long propagation. In addition, when the PDxOs were reimplanted in mice their tumor growth rates were not statistically different from the growth rates of the parental PDX, even when implanted after different time points in culture.

Given the organoid relevance to cancer research, large efforts are undertaken to make PDOs available to the scientific community. The first was the establishment of a large PDO collection, the Hubrecht Organoid Technology (HUB) living biobank [123], resulting from the collaboration between the US NCI, the UK Wellcome Trust Sanger Institute and the HUB foundation, known as the Human Cancer Models Initiative. The HUB biobank contains and generates hundreds of PDOs from healthy and tumor tissues together with baseline clinical data from the original patients that are accessible for both industry and academia. 

## 3. Efficacy Assessment in 3D In Vitro Cancer Models

An accurate quantification of the anticancer efficacy of a drug or a therapeutic intervention is a critical aspect of the development process. However, in the context of the 3D in vitro cancer models, there are no well-established and standardized approaches to evaluate the response to an anticancer treatment [16,124]. As a result, researchers can apply a variety of different assays and techniques to characterize the anticancer drug efficacy [61], including assays evaluating cell viability, proliferation or apoptosis as well as approaches based on microscopy imaging. Many of these techniques are common to both spheroids and organoids. However, some assays are more frequently used and have to be preferred over others, depending on the final scope of the study, on the 3D culture system under consideration, and on the characteristics of the cells composing the system [125,126,127].

In this review, we will focus on assays based on cell viability and on microscopy imaging. For both of them, we will shortly describe the main characteristics, report the most relevant applications stratified in spheroids and organoids, and, lastly, introduce the efficacy metrics typically derived from these measurements.

### 3.1. Cell Viability-Based Assays

Cell viability assays evaluate the well-being of cells in response to drug exposure by measuring the proportion of living cells or of their fluorescent/luminescent signals. They can be classified in dye exclusion assays, colorimetric assays, fluorometric assays and luminometric assays [128] (see Table 4). Dye exclusion assays use dyes to differentiate between live and dead cells, exploiting their different membrane permeability. Indeed, live cells have intact membranes that prevent the dyes from entering, while dead cells do not present this barrier, and so allow the dye passage [129]. Colorimetric assays measure the metabolic activity of cells by detecting color changes of a specific compound. These assays use reagents that undergo a measurable color change in the presence of live cells as an indication of their biochemical activity [130]. Fluorescent viability assays utilize specialized dyes or probes that emit fluorescence when exposed to certain wavelengths of light. These assays are relatively simple to perform and can be more sensitive than colorimetric assays in detecting subtle changes in cell viability [131]. Finally, luminometric assays measure the amount of light emitted by cells, based on the principle that living cells have an active metabolism and produce a constant amount of ATP [132].

Cell viability assays generally require the destruction of the 3D in vitro models. Consequently, they are classified as endpoint assays, as they provide a snapshot of what is occurring at a single time point and do not capture the entire time course of drug response. Because the response to treatment could vary over time, the results of cell viability assays could be dependent on the time at which they are performed. This could lead to misspecifications of treatment effects, which could be especially relevant when the time required to obtain a response to treatment is not yet well-established or when some resistance mechanisms arise during drug exposure.

#### 3.1.1. Spheroids

A variety of techniques and assays to assess the cell viability in cancer spheroids has been used. Most of them are derived from the methods applied for the traditional 2D in vitro cell cultures with no or only minimal modifications. For example, Kochanek et al. used the fluorometric CellTiter-Blue assay to quantify the effects of 19 different approved anticancer drugs on spheroids derived from five head and neck squamous cell carcinoma cell lines [143]. Kessel et al. performed a fluorimetric assay using the Calcein AM, propidium iodide and Hoechst 33342 to determine cell viability of glioblastoma spheroids derived from the U87 cell line and treated with Tanespimycin, Paclitaxel, Temozolomide or Doxorubicin for drug-screening application [83]. Differently, a dye exclusion assay, the Trypan Blue, was used by Xu et al. to assess the effectiveness of Atorvastatin in combination with Celecoxib and Tipifarnib on spheroids derived from the pancreatic cancer Panc-1 cell line [133].

However, because these assays were originally designed for 2D monolayer cultures and used outside their original intended use, they could not be efficient when applied to spheroids, due, for example, to decreased penetration of dyes/reagents in large 3D structures, decreased lytic activity due to the presence of an extracellular matrix and the tight cell–cell junctions of the 3D cellular aggregates [132]. Therefore, the application of a 2D viability assay to 3D in vitro cancer models requires an accurate optimization of the viability protocol as well as an extensive validation of its accuracy. For example, Piccinini et al. compared the repeatability and the reproducibility of viability results obtained through the Trypan Blue assay on 2D cell cultures and 3D spheroids from two different cancer cell lines (the A549 lung carcinoma and PANC-1 pancreatic carcinoma cell line) [152]. Viability measurements independently obtained by two biologists who analyzed 105 different samples were compared, highlighting an approximate variability of 5%, similar for 2D and 3D cultures. Further, Eilenberger et al. optimized the standard alamarBlue viability protocol for the application to spheroid cultures to enhance the assay precision [153]. Key modifications involved the increase of the incubation period from the original 2–3 h to 24 h. Cell viability of 2D cultures and spheroids from the HepG2 liver cancer cell line exposed to sorafenib treatment was measured and compared. For spheroids, an extended incubation period resulted in a tremendous increase in the assay precision with an overall reduction of the standard deviation range to 4–10%. In comparison, 2D monolayer cultures displayed a similar comparable precision and reliability for any alamarBlue incubation time.

To overcome the limitations of 2D-derived viability assays, methods specifically designed for the 3D in vitro systems were developed. Among the commercially available assays, one of the most widely used is the CellTiterGlo-3D (CTG-3D), an ATP-based luminescent assay, whose application is documented for spheroids derived from different cancer types and cultured with different techniques [144,145]. Few works compared viability measurements obtained with 2D-derived and 3D-specific assays, demonstrating that methods specifically designed for 3D in vitro models generally perform better. For example, Zanoni et al. tested the Trypan Blue assay and two 3D-specific viability assays, including the CTG-3D, to evaluate the effect of increasing concentrations of an anticancer agent on large spheroids [64]. All the assays showed a decrease in cell viability in treated spheroids compared to controls. However, the Trypan Blue assay failed to capture the dose-dependence of drug effect and exhibited a high variability of viability results (CV = 42.7). Differently, the two 3D-specific assays correctly identified the dose-dependent treatment effect and showed a similar and low variability (CV = 7.53 and 7.23). Further, Dominijanni et al. assessed and compared the accuracy of various commercially available cell viability assays developed for 2D or 3D applications (including MTS and CTG-3D) on different 3D hydrogel constructs derived from the HCT-116 human CRC cells [132]. An important variability in the viability results was found, with CTG-3D exhibiting the most accurate readouts overall regardless of construct size, cell density and hydrogel makeup.

Despite their popularity, the viability-based methods are unable to fully grasp the whole spectrum of spheroid responses to treatment. For example, morphological changes of the 3D spheroid structure induced by the treatment cannot be captured [143]. Consequently, measuring cell viability alone might provide an incomplete picture of the drug impact on spheroids, potentially resulting in an underestimation of the actual drug efficacy.

#### 3.1.2. Organoids

Cancer organoids exhibit a more complex morphological structure than cancer spheroids, further challenging the assessment of cell viability. As a result, the use of specialized viability assays, specifically designed for 3D in vitro models, is of great relevance to accurately assess the overall viability of organoid cultures. The previously introduced CTG-3D luminescent assay is the most widely used method in organoid studies [148,149,150,151].

Despite this consideration, the use of viability assay techniques not specifically designed for 3D in vitro models is still documented in some organoid studies. For example, Mazzocchi et al. used the dye exclusion assay based on calcein-AM and ethidium homodimer-1 [141], while Fusco et al. used the Trypan Blue assay [135]. Such examples are, however, less frequent and popular compared to spheroids.

As for spheroids, the use of viability-based methods for the drug efficacy assessment presents some limitations also when applied to organoid cultures. In addition to previously discussed issues, viability assays provide only a well-level quantification of organoid response to treatment and fail to capture the contributions of inter- and intra-heterogeneity of PDO cultures. Consequently, the presence of cell subpopulations with varying drug sensitivities cannot be detected through cell viability assessment [127,154]. Additional techniques, such as imaging-based assays, must be used to better understand the heterogeneity of cancer organoid response to drug treatment.

#### 3.1.3. Efficacy Metrics

**Figure 3:**
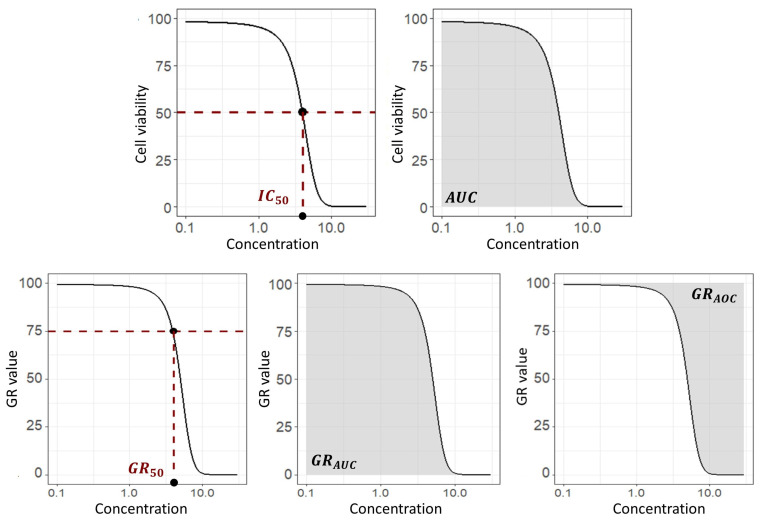
Efficacy metrics derived from cell viability-based assays. In the upper panels the IC50 and AUC metrics from the concentration–viability curve; in the lower panels the GR50, GRAUC and GRAOC metrics from the concentration-growth inhibition growth curve.

Cell viability is typically measured at multiple concentrations of drugs or treatments and used to build the concentration–response curve, illustrating the relationship between the drug concentration and the corresponding percentage of cell viability compared to the control, i.e., untreated culture. From the obtained concentration–response curve, different parameters can be derived to quantify the potency of drug treatment (see Figure 3). The most popular metrics of drug efficacy are the half-maximal inhibitory concentration, IC50, and the area under the curve, AUC [155]. The first one represents the concentration of drug required to reduce the cancer cells by 50% with respect to the control. It is a simple and easy-to-understand parameter that, providing a clear threshold value to discriminate between effectiveness or ineffectiveness, allows a quick comparison of different drugs or treatments. However, IC50 accounts for the effect of a drug or treatment only at a single concentration point, without considering the anticancer activity at different concentrations. Conversely, AUC, measuring the area under the concentration–response curve, integrates results across a wide range of drug concentrations, thereby providing a more comprehensive picture of the overall drug effectiveness. However, AUC is a more complex metric that can be difficult to interpret and compare across different drugs or treatments and does not provide a clear threshold value to discern between effective or ineffective treatment [156].

It has been demonstrated that IC50 and AUC are both influenced by the growth rate of cancer cells. For example, rapidly growing cells may require a higher drug concentration to be reduced by 50% than slowly growing cells. To account for this issue, in 2016 Hafner et al. proposed to replace the concentration–viability curve with the concentration–growth rate inhibition curve [157]. First, the growth rates are simply computed from endpoint measurements of cell viability in treated and untreated samples, given the initial cell number. Then the growth rate inhibition, GR, is defined as the ratio between growth rates under treated and untreated conditions, normalized to a single cell division. The corresponding efficacy metrics are the concentration at which the growth rate is inhibited by 50%, GR50, the area under the concentration–GR curve, GRAUC, and the area over the concentration–GR curve, GRAOC (see Figure 3).

Finally, it is important to underline that all the previous efficacy metrics inherit the limitations of the viability assays from which they are derived. In particular, they are dependent on the time at which the viability assays are performed. Therefore, if the response to treatment varies over time, the efficacy metrics based on cell viability assays could be not suitable to quantify the treatment effects on spheroids or organoids. In addition, this time-dependence hampers the comparison across different studies if the assays are performed at different time points.

### 3.2. Image-Based Assays

Image-based techniques provide alternative tools to evaluate the effects of anticancer treatments [158], in particular on 3D in vitro cancer cultures. These techniques allow the size, morphology and metabolic status of 3D in vitro cancer models to be monitored at multiple time points, thus enabling real-time assays of responses to treatment exposure. Several imaging techniques are available, ranging from the simplest and cheapest brightfield microscope analysis to complex and expensive advanced microscopy techniques. 

Each technique is characterized by its own advantages and specific applications (see Table 5). Brightfield and fluorescence microscopy can be used to analyze the structure and cell organization of the 3D in vitro cancer models and of their surrounding TME [159]. Fluorescence microscopy combined with suitable cell labeling can allow the assessment of cell viability and proliferation. Confocal microscopy can create high-resolution images of thick samples, providing information on the internal 3D structure. Live-cell imaging allows to monitor the cell activity in real-time. Super-resolution microscopy provides detailed images of cancer cells and their microenvironment. Additionally, other imaging-based methods, such as automated imaging, label-free biosensors, and imaging mass cytometry, can provide insights into different aspects of cancer biology and drug activity [160].

Microscopy techniques have to be coupled with appropriate software for image-data processing. Depending on the nature of the 3D in vitro model, on the images to process and on the level of accuracy and precision required, several specialized tools are available, ranging from simple image processing software to more advanced algorithms. There are both commercial products, such as Imaris (Bitplane AG, Zurich, Switzerland) and Amira (Visage Imaging Inc., Carlsbad, CA, USA), and open-source solutions, such as ReViSP [161] and its updated version ReViMS [162], OpenSegSPIM [163] or OrganoID [164].

**Table 5:** Image-based assays commonly applied in spheroid and organoid studies.

Type of Image-Based Technique	Monitored Quantity	Applications
Brightfield imaging	Morphology, dimension	Spheroids [165,166] Organoids [148]
Fluorescence imaging	Dimension, viable cells, cell density	Spheroids [143,167] Organoids [168]
Confocal live cell imaging	Morphology, internal 3D architecture	Spheroids [169] Organoids [170]
Live cell imaging	Cell activity in real-time, morphology, dimension	Spheroids [171] Organoids [170]
Optical metabolic Imaging	Fluorescence intensity and lifetime of NADH and FAD	Organoids [154,172,173]

Image-based approaches to analyze 3D in vitro cancer models is a wide research field under further development, and currently, there is no gold standard. A comprehensive review of all the available techniques and applications is out of the scope of this paper. In the following, we will report an overview of the most relevant and frequently applied approaches.

#### 3.2.1. Spheroids

In spheroid studies, the applications of image-based techniques typically aim to monitor the temporal dynamics of the 3D morphology over several days in order to identify possible changes induced by the treatment. To this end, multiple morphological features, such as the volume, shape, perimeter, density and diameter, are tracked before, during and after drug exposure. Symmetrically to the in vivo settings, a reduction of spheroid volume is generally considered as the primary endpoint of treatment efficacy [64]. Therefore, tracking the temporal dynamic of spheroid volume is a crucial task. 

The selection of the imaging techniques depends on the spheroid morphology and on the measurement accuracy that is considered acceptable. 2D brightfield imaging, coupled with appropriate software for image-data processing [161,165], could be sufficient to accurately reconstruct the 3D structure of sphere-like spheroids. Instead, for more irregular structures, advanced 3D imaging systems, able to acquire data of the whole 3D surface, are needed to obtain reliable results. A study by De Santis et al. compared the performances of different open-source software in calculating spheroid volume from light-sheet fluorescence microscopy images, with the aim of providing guidelines for researchers on which is the “best software” according to the characteristics of the 3D in vitro model to analyze [174].

Several applications of image-assay techniques in spheroid studies can be found in the literature. In the study of Takuri et al., an inverted fluorescent microscope was used to evaluate the volume changes in CRC spheroids treated with four different drugs [167]. The PrestoBlue cell viability reagent was used to stain the living cells, and the intensity of fluorescent signal was monitored through microscopy imaging and used to derive spheroid volume based on the strong correlation between volume and cell viability. Another interesting example is provided by the study of Kochanek et al. [143], in which the treatment effects on spheroids from head and neck squamous carcinoma were assessed by using various techniques, including an automated imaging system that tracks changes in the spheroid shape, perimeter, density and diameter on a daily basis. Further, Chen et al. investigated the treatment impact on breast cancer spheroids by monitoring their growth over a period of 30 days [166]. The spheroid size and volume were determined through a combination of brightfield microscopy and ImageJ software (National Institute of Health, USA). Finally, in the study of Rodallec et al. [175], a fluorescent microscope was used to monitor cell growth in untreated and treated spheroids generated from bioluminescently-labeled breast cancer cells. Microscopy imaging was used to quantify the antiproliferative activity of investigated treatments that resulted, depending on cell type, treatment schedule and spheroid size.

#### 3.2.2. Organoids

Image-based assays play a crucial role also in efficacy assessment in cancer organoids. The imaging techniques used to monitor the treatment response of organoids are similar to those applied to spheroids. For example, Yao et al. used an inverted microscope in a brightfield setting to monitor the temporal size changes of 80 PDOs derived from patients with locally advanced rectal cancers [148]. Images were taken every three days for a 24-day period after drug treatment and analyzed using Image-Pro Plus 6.0 (Media Cybernetics, Inc., Rockville, MD, USA), a commercial dedicated software. The areas of all the living 3D structures composing the PDOs were derived and gathered to characterize the organoid size at well-level. This method was found to be as informative as monitoring cell viability over the same time period. 

However, it is important to underline that image analysis of cancer organoids is more challenging compared to spheroids due, for example, to the presence of multi-objects per well, their movements across focal planes as well as their heterogeneity in size and shape, which can be particularly irregular. Hence, advanced imaging approaches could be necessary for a robust quantitative analysis of organoid cultures. For example, Z-stack technology, i.e., a continuous scan of the same field at different Z-axis levels and merging of the images from different layers into a final one, could be fundamental to capture all the 3D structures composing the organoid culture and gather their maximum cross-sections into a single image [176].

Single-object tracking is also crucial to account for the intra-organoid variability of size, shape, growth pattern and response to treatment and, thus, to overcome the limitation of a well-level quantification of treatment efficacy. To this regard, Skala et al. showed that normalizing growth to the pretreatment size for each organoid structure decreased the measurement noise, highlighting the importance of monitoring each organoid individually [127]. Kim et al. proposed a very complete quantification workflow, based on dynamic confocal live cell imaging, to monitor tumor growth and drug response in CRC PDOs at single-object level [170]. Organoids were fluorescently labeled with Lentivirus H2B-GFP and imaged with DRAQ7 vital dye across multiple time points during drug treatment to track cell birth and death events in the individual 3D organoid structures. From the same images, they derived morphological features of the 3D objects, including volume, sphericity and ellipticity, using Imaris. A strong correlation was found between organoid live cell count and volume. 

Single-object tracking of organoid cultures across multiple time-lapse images could be labor-intensive to obtain. Indeed, several platforms for organoid image-data processing require manual labeling of all the 3D structures in each image. Conversely, the automatization of the tracking process is often based on the labeling of the cellular nuclei, which increases experiment time and complexity and may modify cellular dynamics. Implementing machine learning algorithms could significantly reduce the time needed for the image analysis. For example, OrganoID is a software platform based on a convolutional neural network that is able to identify and track the individual 3D structures composing organoid cultures from a wide range of cancer tissue types in time-lapse imaging experiments [164].

Finally, temporal monitoring of organoid size and morphology could not be able to account for additional treatment-induced effects. For example, drug exposure may cause changes in the cellular composition of organoids that are not reflected by alteration of growth or morphology [86]. The Optical Metabolic Imaging (OMI) provides 3D imaging of single cell-metabolism, thus representing a particularly attractive approach to track the metabolic response to treatment within PDOs at both single-cell and single-object level. It exploits the natural cellular autofluorescence of NAD(P)H and FAD through the optical redox ratio, defined as the ratio between NAD(P)H and FAD fluorescence intensity [172,173]. The OMI method has been recently applied in some PDO studies [154,172,173,177]; among them, a good representative example is provided by Pash et al., who assessed both the changes of organoid size and optical redox ratio [177].

#### 3.2.3. Efficacy Metrics

A variety of efficacy metrics can be derived from results obtained with image-based assays. Among them, the difference in growth rate between treated and untreated 3D structures is the most commonly used. This metric has already been introduced based on cell viability assays, where GR has been defined from viability assessments at a single time point. In contrast, in the context of image-based assays, the growth rate inhibition is computed from multiple measurements in both untreated and treated cultures and can be defined based on several morphological parameters, such as volume, surface area or live cell count. For example, Kim et al. compared growth rates derived from volume and surface area with those computed from the live cell numbers in treated and untreated CRC PDOs [170]; a good consistency was found, although the volume–growth rate had a slightly better correlation.

The growth rate inhibition metric is commonly used in both cancer spheroid and organoid studies. However, for the second application it is important to keep in mind that growth rate differences could not be due to drug treatment effects but can result from the inter- and intra-organoid variability of the growth pattern. In the organoid studies including the single-object tracking, the distributions of morphological parameters and/or of the optical redox ratio in untreated and treated organoid cultures can be considered and compared [177,178], allowing the intra-organoid variability in both tumor growth and treatment response to be accounted for. The Glass’s Delta index [179] can be computed to summarize the treatment effect. It is defined as the mean difference between the treated and control group divided by the standard deviation of the control group, thus considering the treatment change as a function of the distribution of the control organoids. 

## 4. Are 3D In Vitro Cancer Models Good Predictors of In Vivo Anticancer Drug Efficacy?

**Figure 4:**
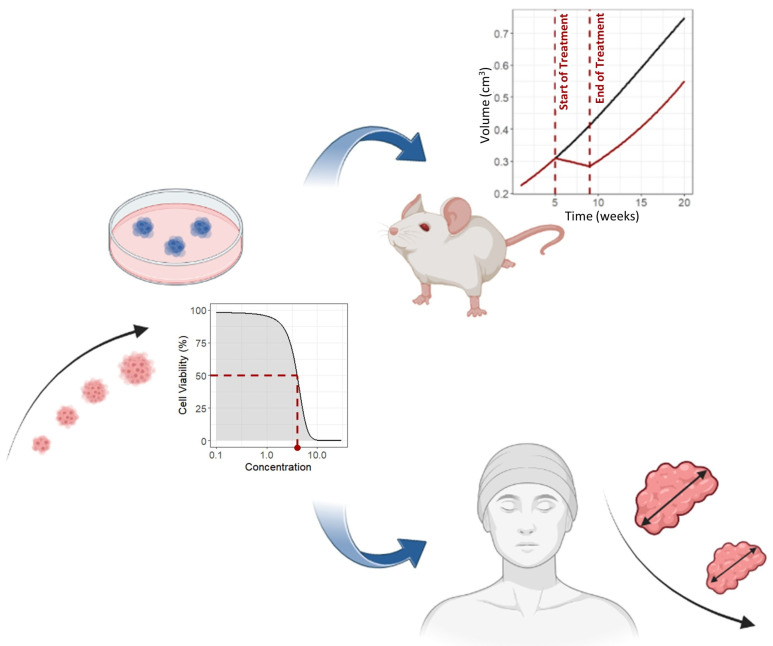
Assessment of predictive value of treatment response in 3D in vitro cancer models for the in vivo (animal and human) response.

The relevance of spheroids and organoids as preclinical models for anticancer efficacy testing is determined, at least partially, by the extent to which the 3D in vitro results can be extrapolated to the in vivo situation (Figure 4). Therefore, a rapidly increasing number of studies are addressing the potentiality of the 3D in vitro models, and especially of PDOs, as predictive tools for in vivo response to anticancer drug treatment. 

In this section, we will provide an overview of the current evidence on transferability of the 3D in vitro results to the in vivo, animal and human, settings.

### 4.1. Spheroids

Several studies have investigated the treatment responses in cancer spheroids compared to 2D cell cultures, highlighting significant differences when the same cancer cell lines were treated with the same compounds in 2D or 3D conditions [139,142,180,181,182,183]. According to the majority of the literature reports, many anticancer compounds lose efficacy in spheroids compared to cell monolayers [63,142,180,181], for example due to insufficient drug penetration into the inner core of the 3D structures. However, this observation cannot be generalized. Indeed, it has been shown that in some cases spheroids can be more sensitive than 2D cell cultures due to the drug-specific mechanisms of action [42,139,183]. For example, it has been reported that kinase inhibitors are more effective on spheroids, while cell cycle inhibitors are more effective on 2D in vitro models [8,42,139].

Based on disparity in testing outcomes between spheroids and 2D cell cultures, many studies concluded that drug response in spheroids better predict the in vivo efficacy, compared to those of 2D cultures. However, only a few studies included an actual in vivo validation of the spheroid results by performing confirmatory xenograft experiments [47,74,75,175,184,185,186].

For example, in the work of Erlichman et al., doxorubicin cytotoxicity was assessed on the MGH-U1 human bladder carcinoma line grown as monolayer, spheroids, and as xenografts in immunodeficient mice [185]. The MGH-U1 spheroids exhibited five-fold resistance to doxorubicin compared to 2D cell cultures, due to the limited drug penetration in the inner core. Indeed, as demonstrated by fluorescence analysis, cells near the spheroid surface were more sensitive to doxorubicin, while drug resistance increased through the inner layers of the spheroids and became maximal near the necrotic core. Doxorubicin treatment of MGH-U1-bearing xenograft mice resulted in low cytotoxicity, consistent with the spheroid model that more closely predicts the in vivo effects than monolayer culture.

More recently, Brodeur et al. conducted a comparative study to investigate the carboplatin response in epithelial ovarian cancer cell lines grown as 2D monolayers and 3D spheroids (and also as ex vivo system, i.e., 3D micro-dissected tumors) and compared them to the in vivo response in xenograft mice [186]. Six cell lines were considered and classified as “sensitive”, “intermediate” or “resistant” based on chemosensitivity to carboplatin quantified through IC50 metrics in in vitro models and TGI in in vivo models. In vivo results from the mouse models correlated with 2D cell culture and 3D spheroid responses in 3/6 and 4/6 cell lines, respectively. This study suggested that the carboplatin response of 3D in vitro models was in line with in vivo results even if 3D spheroids demonstrated higher carboplatin resistance compared to xenograft mice in two cell lines, OV4453 and OV4485.

Another interesting study, evaluating the ability of 3D in vitro spheroids to predict in vivo efficacy in xenograft mice, was conducted by Rodallec et al. [175]. In this work, the effect of a new candidate immunoliposome combined with trastuzumab was evaluated in spheroids generated from two HER2-positive breast cancer cell lines (MDA-MB-453 and MDA-MB-231) before testing it in vivo. Fluorescence microscope imaging was used to monitor cell growth in untreated and treated spheroids and, thus, to assess antiproliferative efficacy of the treatments. Antiproliferative activity resulted dependent on cell type, spheroid size and treatment scheduling, and showed that immunoliposomes performed better (higher cell growth reduction) than current anti-HER2 breast cancer strategies. Confirmatory experiments were then performed in mice orthotopically xenografted with cells from the same cancer lines. A higher efficacy in terms of TGI and prolonged survival was obtained with immunoliposomes compared to two reference treatments, demonstrating the predictivity of 3D spheroids when testing nanoparticles in experimental oncology [187].

Finally, in some studies patient-derived spheroids were developed to predict the patient responses to treatment at individual level. Clinical validation of the in vitro results is reported only in a small set of studies [184]. As an example, in the work of Raghavan et al., spheroids were generated using primary ovarian cancer stem cells derived from three patient ascites and treated with cisplatin alone or in combination with a novel investigated compound [47]. Spheroids from distinct patients showed different responses to drug treatment (based on cell viability at 72 h after drug treatment) that correlated with patient clinical history for platinum response. In addition, two spheroid models were used to initiate tumors in mice. Spheroid-derived xenografts revealed similar responses to chemotherapeutics to the corresponding 3D in vitro model, demonstrating the predictive potency of spheroid-based therapeutic assays compared with those conducted in vivo in spheroid-derived xenograft models.

### 4.2. Organoid

A rapidly increasing number of studies investigated the PDO ability to predict therapeutic efficacy in patients by comparing PDO treatment data with clinical responses of the original patient donors. Although these studies were typically designed with the primary aim of personalized medicine, they provided strong evidence of PDO translational potential. These works were already systematically reviewed in [188,189,190], resulted to be heterogeneous for a multitude of factors. They differed in (i) investigated tumor type and anticancer treatments, (ii) patient cohort size, (iii) study design, (iv) definition of treatment response in PDOs as well in patients, (v) inclusion of parallel PDXs, and (vi) strength of the applied statistical methods and correlation significance. The majority of these studies only provided a descriptive comparison between the organoid and clinical response, generally based on extremely small patient cohorts [188]. Few studies, to date, have performed a rigorous and quantitative clinical comparison that resulted in a statistically significant correlation and/or predictive value [116,148,150,191,192].

The first attempt to rigorously demonstrate the predictive potential of cancer PDOs was provided by the work of Vlachogiannis et al., where the effect of a library of anticancer agents in PDOs (*n* = 21) derived from heavily pretreated colorectal and gastroesophageal cancer patients were compared to their clinical response [191]. The authors found 100% sensitivity, 93% specificity, and 88% and 100% positive and negative predictive values, respectively. However, the criteria to define responses in PDOs and patients were not specified in the work that was carried out retrospectively.

Ooft et al. reported the results of a prospective observational study (the TUMOROID trial) investigating the use of cancer PDOs as a predictive test for chemotherapeutic regimens, including 5-FU in combination with oxaliplatin (i.e., FOLFOX) or irinotecan, or irinotecan alone, in metastatic CRC patients [150]. The temporal dynamics of PDO areas were monitored through microscopy imaging. The inhibition of growth rates after six days of treatment were derived and correlated to the best RECIST response in patients considering the lesions from which PDOs were obtained. Organoids (*n* = 22) correctly predicted the response to irinotecan-containing therapies (irinotecan single or combination treatment) in more than 80% of patients. However, PDOs failed to correctly predict outcomes of FOLFOX treatment for which no correlation with patient responses was found. The absence of correlation for oxaliplatin-based therapy was confirmed also by the APOLLO trial in metastatic CRC [193], where FOLFOX sensitivity in nine PDOs (quantified by the AUC derived from the concentration–viability curve) failed to clearly separate patients who had disease control (i.e., partial responses or stable disease) versus progressive disease (PD). Conflicting results were reported in the work of Ganesh et al., in which the AUCs for FOLFOX (and 5-FU) treatment in seven CRC PDOs correlated well with progression-free survival (PFS) in the corresponding patients (Spearman r = 0.86 and *p* = 0.024) [51].

The phase III CinClare trial, evaluating the predictive value of PDO response to neoadjuvant chemoradiation (i.e., radiation combined with 5-FU with or without irinotecan) in 80 locally advanced rectal cancer patients [148], represented an exception to the generally small size of patient cohorts. PDOs were separately treated with radiations, 5-FU or irinotecan and the least of the ratios of PDO size changes at day 24 to day 0 after treatments was used as predictor of clinical outcome (tumor regression grade upon resection). Of the patients, 85% (*n* = 68) achieved a good clinical response and, accordingly, the parallel organoids were sensitive to at least one of the three treatment components; nine patients had a poor response even if the matched PDOs were sensitive to one or two of the treatment components, and three patients had a good response while the matched organoids resulted resistant to all the treatments. Overall, the combined PDO data highly correlated with patient clinical outcomes, with 84.43% accuracy, 78.01% sensitivity and 91.97% specificity.

The HOPE trial was a prospective clinical study aiming to generate PDOs from PDAC patients and to evaluate the correlation between their drug sensitivity and clinical outcomes [194]. Sensitivities to gemcitabine, 5-FU, oxaliplatin, irinotecan, paclitaxel and other drugs were quantified in 12 PDOs, based on cell viability. Independent AUC assessments (*n* = 49) were calculated for each tested drug and PDO line. For each patient, normalized AUCs were then compared to develop a personalized drug rank that resulted consistent with clinical responses. In addition, a method for classifying PDOs as sensitive or resistant to chemotherapy combination regimens was developed with predictive purpose. PDO AUCs were annotated with clinical outcomes, matching the lowest AUC of the combination with patient response, i.e., disease control or PD. A break of 0.56 segregated PDO AUCs matching to patient disease control from the ones matching PD. Accordingly, organoids were classified as “sensitive” to a combination treatment regimen if at least one of the drugs in the regimen yielded an AUC < 0.56, and “resistant” if all of the drug components yielded an AUC ≥ 0.56.

A different predictive score for response to combined regimens was proposed and validated by Beutel et al. on pancreatic cancer PDOs [192]. Single chemotherapeutic agents (gemcitabine, 5-FU, oxaliplatin, irinotecan, paclitaxel) were separately tested in PDOs, and AUCs for each compound were derived. AUCs were then classified into three groups (high, intermediate and low response) and a score was assigned to each of them (1 = high, 2 = intermediate, 3 = low). For each PDO and tested combination, the mean score was considered as a predictor for the effectiveness of the drug combination: a score ≤ 2 indicated an efficacious combinational chemotherapy, while a score ≥ 2 denoted an inefficacious combination. The comparison with clinical response, classified in disease control or PD, revealed that PDOs successfully predicted outcomes in treatment-naïve patients with an accuracy of 91.1% for first-line (*n* = 11) and 80.0% for second-line regimens (*n* = 5). The prediction accuracy declined to 40% in pretreated patients (*n* = 5).

In the CinClare and HOPE trials as well as in the work of Beutel et al. [148,192,194], the PDO responses to combination therapies were based on the independent assessments of each drug composing the combinations. A different strategy was followed in the work of Witte et al., where the responses to combination treatments were directly evaluated in vitro [116]. In this study, seven PDOs derived from five patients with different subtypes of ovarian cancer were exposed to cotreatment of 5-FU and paclitaxel. Obtained AUCs showed a statistically significant correlation with clinical responses defined based on chemotherapy response score (*p* < 0.001), biomarker normalization (*p* = 0.004) and RECIST (*p* = 0.0092). However, AUCs did not correlate with long-term clinical response (i.e., recurrence and PFS). 

To date, the majority of clinical studies on cancer PDOs were observational and involved standard-of-care treatments. Very few studies involved investigational agents and were of interventional design. For example, Tiriac et al. tested a panel of investigational targeted agents on a set of PDAC PDOs on which all the standard-of-care chemotherapeutics for pancreatic cancer had previously been ineffective [195]. This allowed alternative treatment options to be identified that, however, were not validated in clinics. In the already mentioned APOLLO study, two patients started off-label drug treatment based on PDO results, upon failure of standard treatments; one of them had a partial response [193]. Finally, the SENSOR trial, a prospective intervention trial in metastatic incurable CRC patients which progressed after standard-of-care treatments, aimed to evaluate the feasibility of PDOs to allocate patients for treatment with off-label or investigational agents [196]. In this study, 25 PDOs were treated with five FDA-approved drugs and three agents under development exhibiting substantial responses to one or more drugs in 19 cases. Six patients underwent treatment based on PDO results; none of them obtained significant and durable clinical responses.

Beyond the correlation between organoid and clinical response, some studies investigated the concordance between paired PDO and PDX models derived from the same patients [52,92,98,107,121,197,198]. For example, Schütte et al. collected a large biobank of PDOs and PDXs from CRC patients [198]. Nineteen tumors were modeled in both the systems, allowing a comparative analysis of drug response in PDO/PDX sibling pairs for eight anticancer agents. Treatment outcomes were classified into four categories (strong-, moderate-, minor-response or resistance) based on the IC50 value for PDOs and on relative tumor volume of the treated group versus the matched untreated control in PDXs. Responses were defined to be concordant between PDX/PDO siblings if they did not differ by more than one rank. A general concordance was found, with only two exceptions (AZD8931 and 5-FU). However, comparisons with clinical outcomes were missing.

In a recent study [107] eight PDAC PDOs were treated with commonly used therapeutic agents among which was gemcitabine, showing a different sensitivity than that which resulted from cell viability assessment. The organoid responses to gemcitabine were then examined in vivo through s.c. transplantation of PDOs into immunodeficient mice. Responses to gemcitabine obtained in the PDO-derived xenografts were well correlated with those obtained in vitro, further confirming that organoids can be used to anticipate in vivo drug response. In addition, a library of kinase inhibitors was tested on PDOs allowing a compound able to induce TGI in a gemcitabine-resistant PDO-derived xenograft model to be identified.

Conversely, Guillen et al. investigated the ability of PDxOs to mirror the responses of the matched PDX mice for birinapant, a drug for the treatment of triple-negative breast cancer [121]. Seven patient-derived breast cancers, spanning a range of birinapant sensitivity, were considered in vivo and in vitro. Lines that were shown to be insensitive to birinapant as PDxOs also showed a progressive disease similar to controls in xenograft mice, whereas lines predicted to be sensitive resulted in tumor shrinkage. A similar comparative analysis was performed in the work of Xu et al. [122], where the correlation between the in vitro response in PDxO and the in vivo outcome in PDX was investigated in a large panel of 13 paired PDxO/PDX models across four cancer types for a library of five standard-of-care chemotherapies and seven targeted agents. Drug effects were categorized as either sensitive or insensitive based on the IC50 value in PDxO and TGI in PDX. Statistical analysis of 30 data points indicated that the PDxO in vitro response has an overall good predictive power for the corresponding PDX in vivo outcome (accuracy = 86%, positive predictive value = 75%, negative predictive value = 91%).

## 5. Applications of 3D Cancer In Vitro Models to Drug Development

**Figure 5:**
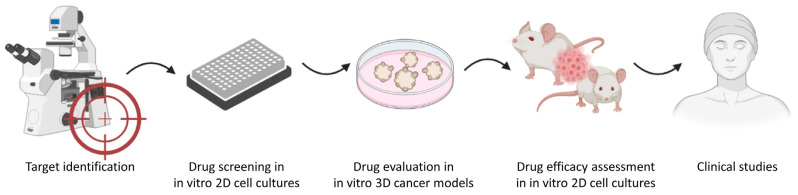
Overview of a possible drug development pipeline that includes the drug efficacy evaluation in 3D in vitro cancer models before in vivo testing in animal models.

The potential of cancer spheroids and organoids to be incorporated into the mainstream development process of new anticancer therapeutics is increasingly recognized, owing to their resemblance to in vivo solid tumors [184,199,200,201]. Despite this trend, their actual inclusion in the assessment of the anticancer efficacy is still sparsely reported [201]. In such examples, in vivo experiments were not completely replaced by spheroids and/or organoids that, instead, were used to screen and further test candidate therapeutics in a more relevant environment compared to 2D cell cultures before starting animal studies [202] (Figure 5). Some interesting applications are reported here.

### 5.1. Spheroids

Cancer spheroids have been available for decades and their potential to either eliminate poor drug candidates at pre-animal stage or to identify promising drugs that had failed in classical 2D cell assays has been widely emphasized [199]. However, to date spheroid experiments have not been yet included in the routinely performed anticancer drug development plans.

One of the most common and relevant applications of cancer spheroids is drug screening. Several works documented the use of spheroids from different cancer types for the selection of the most promising candidates among several new ruthenium complexes, a new generation of metal anticancer drugs which have awoken a lot of interest in the scientific community [203,204,205]. The interest in spheroids for testing ruthenium complexes resulted from their ability to, at least partially, resemble the tumor ECM, which carries out an important role in the activity of ruthenium-derived compounds. For example, De Grandis et al. synthetized a series of novel lawsone-containing ruthenium complexes and screened their antitumor effects against spheroids from the human prostatic DU-145 cells [203]. Anticancer activity was assessed through morphological changes and cell viability, from which the IC50 value was derived. DU-145 spheroids generally resulted in more resistance to ruthenium complexes compared to the 2D-cultured cells. Among the tested agents, complex (4) showed the highest anticancer activity inducing disruption of cell aggregations. Based on the IC50 value, the investigated compound showed a cytotoxic potency 18-fold higher than cisplatin and was selected as a promising candidate for further evaluation. Similarly, Santi et al. evaluated the efficacy of new ruthenium-arene compounds on 3D spheroids of head and neck cancers with or without human papillomavirus infection and compared their effects to the gold standard for this family of compounds [204].

Cancer spheroids also allow fast and affordable large-scale drug screening. Very recently, an automated high-throughput screening of 150,000 compounds in a pancreatic cancer spheroid model directly established from biopsy has been presented and was able to identify leads with potential for further development and clinical applications [206].

In some studies, cancer spheroids have been used to study the mechanisms involved in drug activity or resistance, which also supports the identification of synergistic drug combinations [207,208]. For example, Dubois et al. developed spheroids from two triple-negative breast cancer cell lines (i.e., MDA-MB-231 and SUM1315) and used them to determine the effectiveness of co-treatment with Olaparib and fractionated irradiation with the aim of optimizing the balance between Olaparib cytotoxicity and resistance [146,180,207]. Spheroids allowed long-term culture and, thus, longer exposure time than monolayers (up to 10 days), faithfully mimicking the potential in vivo treatment strategy. Monitoring of spheroid size and metabolic activity revealed a higher efficacy of the low-dose Olaparib compared to the high dose, suggesting the perspective of a low dose and long-term Olaparib administration alongside fractionated irradiation for triple-negative breast cancers.

Co-culture of tumor spheroids with immune cells were recently applied to evaluate the effects of novel immunotherapies [77]. For example, Courau et al. developed a spheroid model by co-culturing CRC cells and immune cells to test the therapeutic potential of immunomodulatory antibodies targeting the NKG2D/MICA-B axis [76]. Treatment enhanced immune-dependent destruction of tumors, increasing immune cell infiltration into tumor spheroids. Results were further validated in clinically relevant 3D in vitro models obtained by co-culturing patient-derived spheroids and autologous tumor-infiltrating lymphocytes from the same CRC patient.

In addition, scaffold-based models, such as embedded-cultures of tumor spheroids within a hydrogel ECM, could provide useful in vitro tools for the evaluation of tumor invasiveness, whose assessment could enhance the spheroid prediction of drug efficiency. For example, Huang et al. developed tumor spheroids embedded within a Matrigel-based ECM to monitor the drug responses of two invasive cell lines from non-small cell lung cancer and CRC to sotorasib (AMG510) under normoxia and hypoxia conditions [209].

Finally, two interesting applications that integrate the use of cancer spheroids for the assessment and the in vitro-to-in vivo extrapolation of anticancer efficacy of new therapeutics in a more complete drug development pipeline were reported [74,75]. In both the studies an investigational compound was evaluated in 3D heterospheroids consisting of pancreatic cancer cells and pancreatic stellate cells (PSCs), before embarking on animal experiments. PSCs have become the therapeutic targets of several novel anticancer strategies, as they are the precursors of CAFs, the most prevalent cell type in the TME and among the major drivers of tumor growth and progression. Schnittert et al. used 3D heterospheroids comprised of PSC and cancer cells from the Panc-1 line to assess the antitumor effect of LXA4, an endogenous bioactive lipid inhibiting the differentiation of human PSCs [75]. In addition, spheroids generated only from Panc-1 cells were considered. LXA4 treatment significantly decreased the size and the growth rate of PSC/Panc-1 spheroids while no effect was observed on the Panc-1 models, confirming that the anticancer activity was due to the PSC inhibition and not to a direct effect on the tumor cells. Based on these findings, the therapeutic efficacy of LXA4 was further examined in a xenograft mice model co-injected with Panc-1 and PSCs, resulting in a highly comparable inhibition profile. Similarly, Kuninty et al. investigated the therapeutic potential of AV3, a novel agent inhibiting PCS activation, in combination with gemcitabine in 3D heterospheroids from co-culture of PSC and Panc-1 or MIA-PaCa-2 tumor cells [74]. Heterospheroids treated with the combination showed a substantial reduction in cell viability, which was much higher than the decrease induced by AV3 or gemcitabine alone. Unlike in homotypic spheroids composed only of tumor cells, adding AV3 did not enhance tumor volume reduction induced by gemcitabine. Spheroid results were then confirmed in co-injected (PSCs + PANC-1 or MIA-PaCa-2) xenograft models and in a PDX of pancreatic cancer. In vivo gemcitabine alone induced a significant TGI, but cotreatment with AV3 reduced the tumor growth more markedly. These two studies provided a further demonstration of correlation between results from 3D in vitro and in vivo models and showed how spheroids can be used to reduce and inform animal studies in a drug development pipeline. 

### 5.2. Organoids

Currently, the major promising applications of PDOs to oncological drug development are (personalized) drug-screening to prioritize candidate agents for in vivo evaluation and drug-gene associations. Indeed, well-established organoid biobanks can be exploited for drug-sensitivity testing, allowing potential active agents to be identified from among newly developed compounds or novel indications for already approved drugs (drug repurposing). In addition, organoid cultures can also be used to investigate the potential beneficials of drug combinations and reversal of drug resistance.

Verissimo et al. first demonstrated the potentiality of PDO libraries in evaluating targeted agents, alone or in combination, in a preclinical setting [210]. They employed a previously established biobank of colorectal cancer PDOs [90] to investigate the effect of multiple clinically advanced targeted inhibitors against the EGFR-RAS-ERK pathway, either alone or in combinations. Based on organoid results, the presence of mutant RAS strongly correlated with resistance to these targeted therapies. In addition, they found a beneficial effect of a combinatorial EGFR inhibition on organoid viability in RAS-mutated cancers, possibly providing an alternative treatment strategy for this subtype of cancer. 

A further proof-of-concept exercise demonstrating that drug sensitivity testing in cancer organoids can inform anticancer drug development was performed in a small library of PDOs from liver cancer patients [92]. Study results provided initial evidence that ERK inhibitors could have a beneficial effect on a subset of liver cancer patients, a therapeutic indication that had not been explored in clinical trials.

Carrera et al. exploited CRC PDOs at different passages to study the effect of plocabulin, a novel antitumor agent of marine origin that was undergoing phase II clinical trials [211]. In organoids derived from three therapy-naive individuals, plocabulin was more cytotoxic than SN38, the active derivative of irinotecan, a chemotherapeutic drug widely used in CRC treatment. Moreover, plocabulin maintained its strong cytotoxic activity in wash-out experiments, where short pulse treatment was as efficient as continuous treatment. Reported results in PDOs reinforced preliminary efficacy evidence from clinical studies, increasing interest in this novel anticancer agent and encouraging further studies.

From previous examples, it is clear that PDOs offer great promise as preclinical cancer models to improve drug development in oncology. However, their actual use is still in its infancy and, as of now, there have been no drugs approved using screening with organoids technology. Only recently, with the simplification of the protocols and high-throughput availability, promising drug candidates have been identified through PDOs [119]. Herpes et al. published the first peer-reviewed work in the oncology field to demonstrate the feasibility of using PDOs to screen a library of compounds and to progress a lead agent from early-stage discovery to clinical trials. More than 500 therapeutic bispecific antibodies were functionally evaluated on a heterogeneous PDO biobank from CRC and paired healthy colonic mucosa samples from the HUB biobank. This led to the identification of MCLA-158, a bispecific antibody that binds the LGR5 marker and the EGFR on cancer stem cells, inducing a robust blocking of growth in organoids. Results obtained in the PDOs were then validated in in vivo models. MCLA-158 induced TGI and damped metastasis formation in organoid-derived PDX obtained by engraftment of PDOs in mice. This promising new agent is currently under evaluation on patients in clinical trials. Notably, the development of the lead agent was accomplished in about five years, a significantly shorter timeframe than a classic drug discovery and development pipeline. 

## 6. 3D In Vitro Cancer Models as an Alternative to Animal Testing: Advantages and Current Challenges

One of the major obstacles in the development of anticancer drugs in a time- and cost-effective manner is the lack of translatability of preclinical results, generally obtained in 2D cell cultures and xenograft models, from bench to bedside. Therefore, there is an urgent need of incorporating more predictive in vitro cancer models throughout the drug discovery and development pipeline to both increase the translational success of preclinical studies, which ultimately results in better treatment options for cancer patients, and to reduce the animal use, in alignment with the 3Rs commitment. 3D in vitro cancer models, in the form of spheroids or organoids, offer a new and exciting preclinical platform potentially able to provide more translatable data to the clinics, while ensuring the 3Rs of animal use. 

Spheroids and organoids have distinct and overlapping features, which result in distinct and overlapping applications. Spheroids are of low complexity in mirroring in vivo tumor organization and generally showed little histological resemblance to the original tumor. However, they faithfully mimic metabolic and proliferating gradients of the in vivo tumors and develop clinically relevant resistance to anticancer treatment. The simplicity and low cost of generation, together with these features, make spheroids an extremely useful model for efficacy testing in drug screening programs. Organoids are more complex in vitro systems that histologically and genetically resemble the original tumors from which they are derived, thereby allowing modeling of the inter- and intra-tumor heterogeneity observed in cancer patients. Organoids can be generated from a very small amount of human tissue, expanded for long-term culture and cryopreserved in biobanks which could serve as a source of biomaterial for world-wise use and be an oasis for rare cancer types. These characteristics allow their use for a wide range of applications, which include anticancer drug efficacy assessment. Moreover, additional TME components can be incorporated in both the 3D in vitro systems using co-culturing techniques, thus providing relevant in vitro tools to test the anticancer activity of agents targeting the stroma cells, including immunotherapy.

Despite the excellent properties of spheroid and organoids, several challenges still hamper their actual use as preclinical tools for therapeutic efficacy evaluation in the development programs of new anticancer agents [59,126].

The first and major issue relates to the absence of standardized culture protocols. The wide variability and inconsistency of methods to generate both spheroids and organoids, together with the often low methodological transparency of published experiments, leads to a lack of reliability and reproducibility of results across studies [59,100,126,212]. For example, culture technique, medium composition and cell seeding density significantly affect spheroid formation, resulting in difficulties in consistently producing 3D structures of homogeneous shape and size [8,59,64]. This represents an important bottleneck for the anticancer efficacy assessment in cancer spheroids, as differences in the morphological features will result in different therapeutic responses. Similarly, the current use of non-standardized and ill-defined culture techniques across PDO studies introduces an additional and confounding source of variability, preventing a faithful representation of the intrinsic cancer biological heterogeneity. The origins of technical variabilities can include cancer tissue sources (primary or metastatic lesions) and subsequent processing techniques, medium formulation as well as heterogeneous and animal-derived matrices [100]. Therefore, there is an urgent need for standardized methods and guidelines and/or for a transparent knowledge base for the generation and culture of both spheroid and organoid models.

Another relevant drawback encompasses the lack of quantitative and robust evaluation methods for drug efficacy [59]. As previously discussed, a plethora of different assays, based on both cell viability and microscopy imaging, have been employed to characterize drug efficacy in 3D in vitro cancer models. Each technique is characterized by its own advantages and limitations, so that a reference has not yet been established. In addition, several techniques have been inherited from 2D in vitro cultures and, although considerable progress has been made to adapt them to the 3D models, many challenges remain to be addressed, including an accurate optimization of the viability protocols as well as an extensive evaluation of their accuracy. Finally, several efficacy metrics used to summarize the response to treatment, such as IC50, AUC, GR50, etc., are strongly affected by tumor growth rate and experimental settings, such as drug exposure duration, concentration level and time at which the assays are performed, thus often providing biased estimates of treatment efficacy.

Additionally, because of these open challenges, the full potentiality of 3D in vitro cancer models to predict the in vivo, in animal and in human, drug efficacy is poorly outlined. Some studies pointed out that spheroids more closely resemble the in vivo treatment response observed in xenograft mice than monolayer cultures, including confirmatory experiments in animals. However, only a qualitative comparison between in vitro and in vivo treatment responses was ever carried out, based on few cancer cell lines and/or drug treatments. A more systematic analysis that includes a wide panel of cancer cell lines and anticancer agents might be needed. Available data on drug sensitivity in the NCI60 cell lines, as both monolayer cultures and in vivo models, could allow for a stronger assessment of predictive power of 3D spheroids in forecasting the in vivo drug efficacy in cancer animal models [63]. Regarding cancer organoids, a multitude of works qualitatively compared the treatment responses in paired PDOs and PDXs with the clinical responses in the original patient donors, highlighting a good consistency. However, the small sample size of the PDO studies together with the incompletely matching conditions, such as tested drug concentrations, exposure duration as well as treatment efficacy criteria, considered in PDOs, PDXs and patients, affected the strength of the results. In addition, the experimental design, the protocols for PDO generation, the assays to quantify treatment efficacy in PDOs and the readouts to define both the in vitro and in vivo responses significantly differed among these studies. This heterogeneity avoids gathering results from different studies and performing a pooled analysis in order to quantitatively derive an estimate of the overall PDO performance in predicting in vivo (in animal and human) responses. More comprehensive studies and quantitative data are necessary to make an accurate assessment of PDO predictivity [188]. Finally, the majority of these studies were performed retrospectively and involved only gold-standard treatments and not investigational agents. Cancer PDOs were generally asked to retrospectively categorize compounds into “active/not-active” as well as patients into “respondent/not respondent”, while their capability in prospectively anticipating the conditions, i.e., dose or concentration levels, needed to achieve a therapeutic effect and to support the drug development process, has not yet been investigated.

When the current issues relating spheroids and organoids are addressed, including standardization of culture protocols and assay techniques, establishment of quantitative evaluation methods for drug efficacy and validation of drug response predictions, these 3D in vitro cancer models will provide a powerful platform for the preclinical evaluation of anticancer drug candidates. The recent passage of the FDA Modernization Act 2.0 [213], which allows for the replacement of certain animal studies by using alternative models such as spheroids and organoids, could cause a surge in the popularity of these 3D in vitro cancer models, leading to an improved standardization of the methodologies [214]. Up to now, it still seems unfeasible that 3D in vitro cancer models could completely replace in a short time in vivo animal models for testing anticancer efficacy. More realistically, in the near future, their use could become a mandatory step between 2D in vitro and in vivo animal models. Identifying and eliminating those treatments that did not show any interesting efficacy in 3D in vitro cultures will reduce animal use and, thus, the relating costs and ethical issues. If the predictive ability of 3D in vitro cancer models was confirmed to be greater than current preclinical cancer models, the integration of spheroid and organoid studies in the drug development pipeline could enhance the transferability of preclinical results from bench to bedside. This could speed up the number of effective candidate drugs that reach clinical development, thereby reducing the number of enrolled patients receiving ineffective treatments and increasing the success rate of clinical studies. In addition, the more easy and cost-effective generation of patient-derived cancer models, such as PDOs, could facilitate an early identification of target patients who benefit most from a specific treatment, moving a step forward to the adoption of a more personalized approach in oncological clinical trials.

## 7. M&S May Enhance 3D In Vitro Cancer Models

M&S may enhance the use of 3D in vitro cancer models in translational oncology, contributing to the establishment of spheroids and organoids as preclinical tools for the assessment of anticancer drug efficacy, in conformity with a model-informed approach to drug discovery and development (MID3) [215,216].

Mathematical models represent the most comprehensive approaches for extracting, summarizing and integrating information obtained in the often less-than-optimally designed experiments performed in the preclinical phase of oncology drug development [217]. Up to now, M&S has been of little impact in the field of 3D in vitro cancer models [218,219,220,221,222].

**Figure 6:**
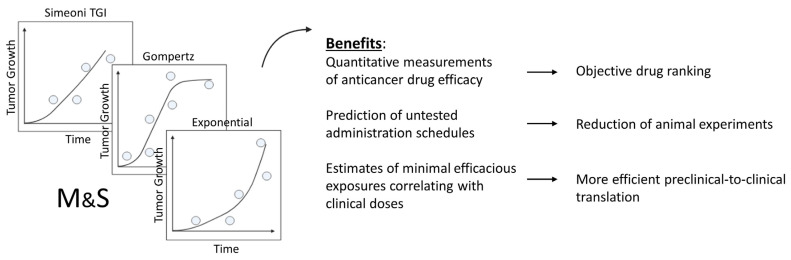
Model-informed preclinical development of anticancer agents.

In contrast, a multitude of mathematical modeling approaches, which describe the anticancer treatment effect on 2D in vitro cell cultures and xenograft animals, have been developed [217], proving an impressive proof-of-concept of the M&S potential to improve the power of preclinical cancer models. In particular, mathematical models quantitatively linking the drug concentration time curve to TGI are of extremely relevant value [223,224,225,226,227]. Among them, the Simeoni TGI model [223] has been applied by several international research groups on a huge panel of xenograft studies as well as in vitro data [228] involving a multitude of different cancer cell lines and anticancer agents, becoming a reference in the field. Several features determined the popularity of this model (Figure 6): (1) it provides quantitative measurements of the anticancer drug efficacy that, differently from simple efficacy metrics directly computed on experimental data (i.e., in vivo TGI percentage), are tumor/compound-specific and independent of experimental conditions (i.e., dose, time and dosing regimen) [229], allowing a drug ranking; (2) it is able to predict outcomes of administration schedules not experimentally tested, reducing in vivo studies; (3) it provides estimates of the minimal drug concentration level needed to guarantee tumor eradication in xenograft mice [230] that strongly correlated to doses administered in patients [231], anticipating the minimal efficacious exposure to be targeted in clinics and supporting the study design of early clinical studies during the drug development process [232,233].

In the field of xenograft experiments, M&S has created a unique opportunity for supporting anticancer drug development. The challenge ahead is now to exploit M&S to address open challenges of 3D in vitro models, thereby boosting and improving their use for anticancer drug activity assessment in translational cancer research [15,212,234]. Mathematical modeling may provide an analysis-assisting tool, filling the lack of a quantitative evaluation method to characterize tumor growth and anticancer drug efficacy in spheroids and organoids. In addition, once adequately qualified, mathematical models may be efficiently used to predict outcomes of conditions not experimentally tested via simulations, reducing wet-laboratory experiments and the associated costs [212]. More relevantly, M&S can increase the quantity and quality of information obtained from spheroid and organoid studies, enabling a more efficient translation of 3D in vitro results to the in vivo settings.

In summary, integration of M&S could significantly contribute to the refinement of 3D in vitro cancer models, increasing their potential to better inform the subsequent in vivo step, in alignment with 3R principles [235].

## 8. Conclusions

3D in vitro cancer models, such as spheroids and organoids, represent a promising preclinical platform for anticancer drug efficacy evaluation that is potentially able to increase the translational success of preclinical studies, thereby resulting in better treatment options for cancer patients, and to reduce the animal use, in alignment with the 3R commitments. Up to now, some issues relating to spheroids and organoids remain unaddressed, including the standardization of culture protocols and assay techniques, the establishment of quantitative evaluation methods for drug efficacy and a complete validation of their predictive capabilities of in vivo treatment response. M&S could significantly contribute to addressing these open challenges, thereby boosting and improving the establishment of the 3D in vitro cancer models as preclinical tools for the anticancer efficacy assessment in translational cancer research.

## Figures and Tables

**Table 1:** Scaffold-free techniques.

Techniques	Description	Applications
Agitation-based	Cells aggregate under continuous stirring to avoid adherence to surfaces and to induce self-assembly.	Spheroids [19,20] Organoids [41]
Liquid overlay	Cells are seeded in low-adhesive surface plates or plates coated with materials that prevent cell attachment.	Spheroids [42,43] Organoids [44]
Microfluidics	Microfluidics are chips composed of microchannels and microchambers where cells suspended in media can circulate and accumulate in the chambers, so forming aggregations.	Spheroids [45,46]
Hanging drop	Cell liquid drops are suspended on a lid that is then inverted; surface tension and gravity induce aggregation.	Spheroids [47,48]
Magnetic levitation	Cells are magnetized through a mixture of magnetic nanoparticles and subsequently incubated under magnetic forces to overcome gravitational force, allowing levitation and, consequently, cellular aggregations.	Spheroids [49]

**Table 2:** Scaffold-based techniques.

Scaffold Type	Description	Applications
Hydrogels	3D polymer networks with high water content, similar in bioactivity, viscoelasticity and mechanical properties to native ECM.	Spheroids [31,50] Organoids [51,52]
Decellularized	Scaffold consists of a decellularized ECM that is obtained from native (or regenerated) tissue by removing the cellular components through physical, chemical and enzymatic means.	Spheroids [53,54]
Fibrous	Fibrous matrix creates an environment that supports the proliferation, growth and migration of cancer cells. The most common technique to obtain nano fibers is electrospinning.	Spheroids [35,55]
Microsphere	Cancer cells are encapsulated in engineered microspheres composed of porous tissue which are geometrically similar in size and shape to tumor spheroids.	Spheroids [37,56]
3D bioprinted	Complex and viable 3D geometric shapes, generated by computer-aided projects.	Spheroids [57,58]

**Table 3:** Success rates for PDO establishment.

Cancer Type	Sample Size	Efficiency	Reference
Bladder	17	70%	[94]
Breast	>100	80%	[93]
Colorectal	20	90%	[90]
	55	~100%	[104]
	8	70%	[105]
Esophageal	32	31%	[96]
Gastric	NR	50%	[95]
Liver	7	100% *	[92]
Lung	23	87%	[97]
Ovarian	33	85%	[98]
Pancreatic	8	80%	[91]
	83	62%	[106]
	19	42%	[107]
Prostate	7	15–20%	[89]

NR = not reported; * tissue samples containing > 5% of proliferating cells.

**Table 4:** Viability assays commonly applied in spheroid and organoid studies.

Type of Viability Assay	Assay	Applications
Dye exclusion assay	Trypan Blue	Spheroids [133,134] Organoids [135]
Colorimetric assay	MTT	Spheroids [136]
Fluorimetric assay	alamarBlue	Spheroids [137,138]
	Calcein AM, Propidium iodide, Hoechst 33,342	Spheroids [83]
	Calcein AM, EthD-1, Hoechst 33,342	Spheroids [139]
	EthD-1, Calcein AM	Spheroids [140] Organoids [141]
	CellTiter-blue	Spheroids [142,143]
Luminometric assay	CellTiterGlo-3D	Spheroids [144,145,146,147] Organoids [148,149,150,151]
